# Do Young People Ever Sit Still? Variations in Accelerometer Counts, Muscle Activity and Heart Rate across Various Sedentary Activities in Youth

**DOI:** 10.3390/ijerph15051009

**Published:** 2018-05-17

**Authors:** Evi van Ekris, Mai J. M. Chinapaw, Joost Rotteveel, Teatske M. Altenburg

**Affiliations:** 1 Department of Public and Occupational Health, Amsterdam Public Health Research Institute, VU University Medical Center, 1081 BT Amsterdam, The Netherlands; m.chinapaw@vumc.nl (M.J.M.C.); t.altenburg@vumc.nl (T.M.A.); 2 Department of Pediatrics, VU University Medical Center, 1081 HV Amsterdam, The Netherlands; j.rotteveel@vumc.nl

**Keywords:** sedentary behaviour, measurement, accelerometry, muscle activity, youth

## Abstract

Evidence of adverse health effects of TV viewing is stronger than for overall sedentary behaviour in youth. One explanation may be that TV viewing involves less body movement than other sedentary activities. Variations in body movement across sedentary activities are currently unknown, as are age differences in such variations. This study examined body movement differences across various sedentary activities in children and adolescents, assessed by hip-, thigh- and wrist-worn accelerometers, muscle activity and heart rate. Body movement differences between sedentary activities and standing were also examined. Fifty-three children (aged 10–12 years) and 37 adolescents (aged 16–18 years) performed seven different sedentary activities, a standing activity, and a dancing activity (as a control activity) in a controlled setting. Each activity lasted 10 minutes. Participants wore an Actigraph on their hip and both wrists, an activPAL on their thigh and a heart rate monitor. The muscle activity of weight-bearing leg muscles was measured in a subgroup (*n* = 38) by surface electromyography. Variations in body movement across activities were examined using general estimation equations analysis. Children showed significantly more body movement during sedentary activities and standing than adolescents. In both age groups, screen-based sedentary activities involved less body movement than non-screen-based sedentary activities. This may explain the stronger evidence for detrimental health effects of TV viewing while evidence for child sedentary behaviour in general is inconsistent. Differences in body movement during standing and sedentary activities were relatively small. Future research should examine the potential health effects of differences in body movement between screen-based versus non-screen based and standing versus sedentary activities.

## 1. Introduction

Children and adolescents spend a large proportion of their day sedentary [1,2] with screen-based activities as the most popular sedentary activities [3,4]. Also, media multitasking, i.e. doing multiple sedentary activities simultaneously, is increasingly common [4]. Overall, based on systematic reviews, the evidence regarding associations of sedentary behaviour and health is inconsistent and varies across different sedentary activities [5,6]. Remarkably, the strongest evidence exists for an adverse association between TV viewing time and health indicators [6]. This may be due to unhealthy eating habits during screen-based activities (including watching food advertisements) or variations in body movement across sedentary activities. Both need very different intervention programs.

We define body movement during sedentary behaviour as the extent to which people move their body while remaining in a seated posture. Examples are fidgeting, movement of hands, arms and legs, and moving on the chair into another seated posture. The extent to which body movement varies across sedentary activities is currently unknown. We hypothesize that (1) TV viewing and other screen-based sedentary activities involve less body movement compared to non-screen-based sedentary activities; and (2) that body movement during sedentary activities decreases with increasing age. Quantifying variations in body movement across sedentary activities is relevant for public health because this knowledge may be valuable for designing interventions: sedentary activities involving the least body movement and highest prevalence may be the preferred target for future health promotion interventions.

Standing interruptions are increasingly used as a potential intervention to reduce prolonged sedentary behaviour in youth [7]. However, experimental studies in adults are inconclusive regarding whether muscle activity and body movement during standing are sufficient to counteract potential adverse cardiometabolic effects of prolonged sitting [8,9]. The extent to which body movement during standing is higher than during sedentary behaviour in young people is currently unknown. This knowledge is required to examine whether replacing sedentary behaviour by standing is likely to result in health benefits relevant to public health.

Indicators of body movement include muscle activity, accelerometer counts and heart rate. Accelerometers are the most commonly used measurement instruments to assess sedentary behaviour and physical activity in field studies. Accelerometers measure acceleration in three planes of motion; the *X*-axis (medio-lateral axis), *Y*-axis (vertical axis), and *Z*-axis (antero-posterior axis) and are most commonly attached to the hip, but the thigh and wrist are used as well [10,11,12,13,14]. These different axes and positions are likely to provide different information on body movement during sedentary behaviour and standing. Muscle activity might be of special interest regarding sedentary behaviour as the detrimental cardiometabolic health effects are hypothesized to result from a lack of muscle activity in weight-bearing muscles [15,16]. Heart rate is a measure of overall body movement and resulting energy expenditure rather than movement of specific body parts.

The present study is the first to examine variations in body movement across a wide range of sedentary activities, assessed by hip-, thigh- and wrist-worn accelerometers, muscle activity and heart rate. Differences in body movement between sedentary activities and standing were also examined. Finally, we examined whether body movement during sedentary activities and standing is different for children and adolescents.

## 2. Materials and Methods

### 2.1. Participants

Fifty-three children aged 10–12 years and 37 adolescents aged 16–18 years participated in this controlled study. Table 1 presents the participant’s characteristics. Participants were recruited from three primary and two secondary schools in or nearby Amsterdam and via announcements on the institute’s website. Participants were eligible in this study when they were apparently healthy; aged 10–12 or 16–18 years old; Dutch or English speaking; and written informed consent was signed by one parent (in case participants were aged <18 years) or the participant (in case participants were aged 18 years). Participants were excluded when they had (1) known physical activity contraindications or (2) major illness/injury (acute or chronic) or (3) physical problems that may limited the ability to perform the experiment. The sample size was based on the COSMIN checklist for measurement properties, with a sample size of 90 participants in total regarded as a good sample size.

### 2.2. Protocol

The study protocol (number 13/031) was approved by the VU University Medical Center Ethical Committee. Participants performed a standardized protocol of approximately three hours (Table 2). The protocol consisted of two non-sedentary activities, namely one standing activity and one dancing activity, and seven sedentary activities: (1) lying down, (2) drawing or writing, (3) playing sedentary computer games, (4) sitting without any additional imposed activity, (5) using a tablet, (6) watching movies, and (7) media multitasking (i.e., playing on a tablet while watching movies). Dancing was included as a control activity in order to examine whether the different devices were capable of distinguishing body movement during higher intensity activity from sedentary activities and standing.

All measurements took place at school or the VU University Medical Center. At each location a large room was set up with nine stations. Per session, a maximum of nine participants were randomly assigned to one of the nine activities as a starting activity. Each activity lasted ten minutes and was followed by a 3–5-min break which included changing to the next station. The start and end times of each activity were recorded. All activities were supervised by trained researchers and video recorded using four cameras. Each session started with attaching devices to the participants, familiarizing them with the activities and informing the participants of the protocol.

### 2.3. Measurements

Participants wore Actigraph *GT3X+* accelerometers (Actigraph, Pensacola, FL, USA) on their right hip and both wrists, attached with adjustable elastic belts. Left- or right-handedness was reported. The Actigraphs were set to collect triaxial acceleration data (X-, Y-, *Z*-axis) at a sampling rate of 100 Hz. Data were downloaded using ActiLife software in 15-s (15-second) epochs.

Participants wore an activPAL^3TM^ monitor (PAL Technologies Ltd., Glasgow, UK) at the front of the left thigh, positioned exactly midway between hip and knee using a tape measure. The activPAL^3TM^ was attached directly to the skin using medical tape. Triaxial acceleration data were collected at a sampling rate of 20 Hz. Data were downloaded using activPAL^3^™ software in 15-s epochs. For the purpose of the present study, we used the triaxial acceleration data of the activPAL^3^™ and not the data of the inclinometer function. The inclinometer function can be used to classify posture (i.e., lying/sitting, standing and stepping).

Heart rate was measured in 15-s epochs using a Polar RS800CX heart rate monitor (Polar Electro Oy, Kempele, Finland) that was attached to an adjustable strap and tied around the chest. Data were downloaded using Polar ProTrainer 5 software (Polar Electro Oy, Kempele, Finland).

Muscle activity of three weight bearing muscles of the right leg (i.e., rectus femoris, vastus lateralis, and gastrocnemius medialis) was measured in a random subgroup (*n* = 38). Participants included in the subsample for muscle activity were not statistically significantly different from participants without data on muscle activity. Muscle activity was measured by surface electromyography (EMG) using the Portilab device (TMS International, The Netherlands). EMG amplitude is a reliable measure of muscle activity [17,18]. Two Ag/AgCl surface electrodes (Blue Sensor, 30 × 20 mm) were positioned on the belly of each muscle and parallel to the muscle fiber direction in a bipolar configuration (inter-electrode distance of 20 mm), according to the SENIAM guidelines [19]. Electrodes for the rectus femoris were placed at 50% on the line from the anterior spina iliaca superior to the superior part of the patella, for the vastus lateralis at 2/3rd on the line from the anterior spina iliaca superior to the lateral side of the patella and for the gastrocnemius medialis at the middle of the muscle belly. A reference electrode was placed on the patella. Electrodes were placed on shaved and cleaned skin. The EMG signal was amplified (20 times), band-pass filtered (10–500 Hz) to remove noise from the signal (e.g., due movement artefacts), analogue-to-digital converted (sample rate of 1000 Hz) and converted to mV on a scale of 0.0175. Subsequently, EMG data were corrected for baseline offset, rectified and summarized into 15-s epochs. For comparison between participants, EMG data were normalized to EMG data measured during a submaximal stepping task, performed at the end of the study protocol. The stepping task consisted of stepping on and off an aerobics step (height: 25 cm) for 1.5 min, at a pace of 114 beats/min (i.e., corresponding with normal walking speed). EMG values during the various activities were expressed as percentage of the 95th percentile of EMG values during stepping. MATLAB (version R2012a, The MathWorks, Inc., Natick, MA, USA) was used for processing of EMG data.

In total, data on 16 body movement variables were obtained: triaxial accelerometer data of the Actigraph positioned on both wrists and the right hip (nine variables); triaxial accelerometer data of the activPAL positioned on the thigh (three variables); heart rate (one variable); and data on muscle activity of three weight bearing muscles of the right leg (three variables). 

Age and gender were collected by child-report. Body weight (kg) was measured to the nearest 0.05 kg with a calibrated digital scale (Seca 861, Seca, Hamburg, Germany). Body height (cm) was measured to the nearest 1 mm using a portable Leicester stadiometer (Invicta plastics limited, Leicester, England). Measurements were taken twice and values were averaged unless the two values deviated by more than one percent, then a third measurement was performed and the deviating value was excluded. Participants wore light indoor clothing and no shoes. Body mass index was calculated (kg/m^2^). Weight status was determined using international Obesity Task Force cut-offs [20].

### 2.4. Data Reduction and Analysis

Before each session, all devices were time synchronized with the system clock of a personal laptop. For each 10-min activity, the last seven minutes of data were included in the analysis ensuring steady state of the activity. This resulted in 28 data points per activity (7 min × 4 data points per minute, i.e., 4 epochs of 15-s). Data from participants deviating from the protocol (*n* = 5) during a specific activity (e.g., standing up during a sedentary activity) were removed. The cleaned data were analyzed in SPSS version 20.0 (IBM Corp., Armonk, NY, USA). Participant characteristics are presented as means with standard deviations. As the activPAL and Actigraph data were zero inflated, medians with 25th percentile (P25) and 75th percentile (P75) were calculated for all body movement variables. Accelerometer data were expressed in counts/15-s, heart rate in beats/minute and EMG values as percentage of the 95th percentile of EMG values during stepping.

Differences between activities were examined by Generalized Estimation Equations (GEE) analysis, a longitudinal analysis technique adjusting for dependency of repeated observations within participants (i.e., 28 data points and nine activities), for which an exchangeable correlation structure was used. For the Actigraph and activPAL data, a negative binomial distribution with log link was used. This type of distribution can be used for count data that are not normally distributed. The corresponding coefficient is the rate ratio for differences between activities. For muscle activity and heart rate, a linear distribution was used. Differences between children and adolescents were examined by including interaction terms in the model. Coefficients are presented with their 95% confidence interval. Statistical significance was set at *p* < 0.05.

## 3. Results

Due to device failure, two participants (one child and one adolescent) had missing heart rate data, one child had missing muscle activity data for the rectus femoris, and one child had missing accelerometer data for the hip and both wrists. Accelerometer data of the non-dominant wrist were excluded for one child because of an arm injury.

Interaction effects for age group were significant in thirteen out of sixteen body movement variables. Therefore, results are presented separately for children and adolescents.

Table 3 and Table 4 show the median values with P25 and P75 of muscle activity, heart rate and accelerometer counts of the hip, thigh and wrists per activity and age group. Table A1, Table A2, Table A3, Table A4, Table A5 and Table A6 in appendix A show the results of the GEE analyses. For all body movement variables, medians and P75 during the different sedentary activities and standing were significantly higher in children than adolescents, while medians and P75 of dancing were higher in adolescents (Table 3 and Table 4). Dancing resulted in significantly higher accelerometer counts regardless of position and axis (Table A1, Table A2, Table A3 and Table A4), higher muscle activity (Table A5), and higher heart rate (Table A6) than all sedentary activities and standing. Results for body movement variables during dancing are therefore only reported in the tables.

### 3.1. Hip Accelerometer Counts 

Medians and P75 of hip-accelerometer counts were zero for most sedentary activities, with few exceptions (Table 3). Standing resulted in median counts higher than zero for the *X*- and *Z*-axis (17 and 26 counts, respectively), and a P75 higher than zero for the *Y*-axis (6 counts). In adolescents, all sedentary activities resulted in median counts and P75 of zero for all different axes. Standing resulted in a P75 higher than zero for the *X*- and *Z*-axis (8 and 13 counts, respectively), but not for the *Y*-axis (Table 4).

In children, computer gaming resulted in significantly lower counts than all other sedentary activities, for at least one of the axes (Table A1). The largest difference was found between computer gaming and just sitting (*Y*-axis, 3.3 times higher) in children, and between tablet use and just sitting (*X*-axis, 3.1 times higher) in adolescents. Counts during the non-screen-based sedentary activities (i.e., just sitting and drawing) were significantly higher than during the screen-based sedentary activities and lying down for at least one of the axes, in children but not in adolescents. Remarkably, in adolescents, drawing resulted in significantly lower counts for the *Y*-axis compared to some of the screen-based sedentary activities. In both age groups, counts were significantly higher during standing than during sedentary activities for the *X*- and *Z*-axis (range: 1.9–12.6 times). For the *Y*-axis, differences were less consistent.

### 3.2. Thigh Accelerometer Counts 

In children, median and P75 thigh-accelerometer counts were slightly higher for non-screen-based versus screen-based activities (Table 3). In adolescents, this pattern was only visible in the P75 (Table 4). In both age groups median, P25 and P75 were higher during standing than sedentary activities.

Counts during non-screen-based activities were significantly higher for at least two axes than during screen-based activities, except for computer gaming, in children (range: 1.5–2.6 times) and adolescents (range 1.4–6.1 times) (Table A2). In children, no differences between screen-based activities were found whereas in adolescents, computer gaming resulted in higher counts than watching movies and media multitasking. In both age groups, counts during lying down and screen-based activities were comparable. Standing resulted in significantly higher counts than all sedentary activities in children (range: 3.2–10.3 times) and adolescents (2.8–13.4 times), except for the *Y*-axis during drawing in adolescents.

### 3.3. Wrist Accelerometer Counts

Medians and P75 of wrist-accelerometer counts for the dominant hand were higher during sedentary activities requiring arm-movement (i.e., drawing, tablet use and media multitasking) than during sedentary activities requiring no arm-movement (i.e., watching movies, lying down, computer gaming and just sitting) in children (Table 3) and adolescents (Table 4), except for just sitting in children. Differences in counts between sedentary activities requiring arm-movement and requiring no arm-movement were less pronounced for the non-dominant hand. In children, standing resulted in higher median counts and P75 than all sedentary activities, except for drawing for the dominant- and the non-dominant hand (Table 3). In adolescents, medians and percentiles for the dominant hand were similar during standing and sedentary activities requiring arm-movement (Table 4).

For the dominant hand, counts during sedentary activities requiring arm-movement were significantly higher than sedentary activities requiring no arm-movement, in children (range: 1.3–8.9 times) and adolescents (range: 1.8–11.6 times) for at least two axes, except for just sitting which was not consistent significantly different from activities requiring arm-movement (Table A3 and Table A4). Drawing resulted in higher counts than all other sedentary activities in children (range: 1.2–8.9 times) and adolescents (range: 1.3–11.6 times).

For the non-dominant hand, drawing was the only sedentary activity requiring arm-movement that resulted in significantly higher counts than the sedentary activities requiring no arm-movement in children (range: 1.3–3.3 times) and adolescents (1.6–3.1 times) (Table A3 and Table A4). Counts during just sitting were significantly higher than all other sedentary activities, except drawing, for at least two axes both in children (range: 1.3–3.4 times) and adolescents (range: 1.6–3.3 times).

For both wrists, standing resulted in significantly higher counts than sedentary activities in children (range: 1.3–9.4 times higher), although differences with drawing were small. For adolescents, standing resulted in significantly higher counts than sedentary activities requiring no arm-movement (range: 2.6–6.5 times) but counts were similar to sedentary activities requiring arm-movement, especially for the dominant-hand.

### 3.4. Muscle Activity

The median muscle activity during sedentary activities was low ranging from 0.9% (media multitasking) to 1.4% (just sitting) of submaximal muscle activity for the vastus lateralis, between 1.7% (tablet use) to 2.8% (computer gaming) for the rectus femoris, and between 1.2% (watching movies) to 1.7% (computer gaming and media multitasking) for the gastrocnemius medialis (Table 3). In adolescents, median muscle activity of the sedentary activities ranged between 0.9% (lying down and drawing) to 1.5% (just sitting) for the vastus lateralis, 1.9% (lying down) to 3.9% (computer gaming) for the rectus femoris, and 0.6% (lying down) to 1.1% (computer gaming) for the gastrocnemius medialis (Table 4). In children, median muscle activity during standing of the vastus lateralis, rectus femoris, and gastrocnemius medialis was 2.4%, 3.1% and 4.4% of submaximal activity, respectively, and in adolescents 3.3%, 4.1% and 3.2%, respectively.

Muscle activity of leg muscles was similar during most of the sedentary activities (Table A5). Differences that were significant ranged from 0.8% to 2.1% of submaximal activity in children and from 0.4% to 3.3% in adolescents. There were no consistent patterns within muscle activity differences between sedentary activities. Muscle activity during standing was not consistently higher than during the sedentary activities.

### 3.5. Heart Rate

Median heart rate during sedentary activities in children ranged from 76 bpm (beats/minute) (lying down) to 84 bpm (drawing and just sitting) (Table 3), and between 69 bpm (lying down) to 82 bpm (just sitting) in adolescents (Table 4). Lying down resulted in the lowest heart rate, followed by watching movies and the other screen-based sedentary activities (i.e., computer gaming, tablet use, media multitasking). Median heart rate during standing was 96 bpm in children and 91 bpm in adolescents.

Heart rate differed significantly between most of the sedentary activities (Table A6) ranging from 1.9 bpm (computer gaming compared to watching movies) to 8.1 bpm (just sitting compared to lying down) in children, and from 2.3 bpm (drawing compared to computer gaming) to 12.8 bpm (just sitting compared to lying down) in adolescents. In children and adolescents, heart rate was significantly higher during the non-screen-based sedentary activities than during the screen-based sedentary activities and lying down. Standing resulted in a significantly higher heart rate compared to all sedentary activities, with differences ranging from 10.6 bpm (just sitting) to 18.6 bpm (lying down) in children, and from 9.4 bpm (just sitting) to 22.2 bpm (lying down) in adolescents.

## 4. Discussion

In both children and adolescents, body movement was generally greater during non-screen-based sedentary activities and standing than during screen-based sedentary activities and lying down. Differences in body movement across various screen-based sedentary activities were generally non-significant, which is in line with previous laboratory studies in children aimed at examining body movement during sedentary versus active gaming [21,22]. Mitre et al. [21] found no differences in energy expenditure and body movement between watching movies and playing sedentary video games, and Straker et al. [22] found no difference in muscle activity of various muscles during watching a DVD and playing sedentary video games. It is therefore unlikely that variations in the association with health outcomes observed for different screen-based sedentary activities (e.g., TV viewing and computer use) in children [6] can be explained by differences in body movement. An alternative explanation may be differential interactions with eating behaviour [23]. Especially TV viewing, for which the strongest evidence for health effects is found [6], is related to the intake of energy-dense foods and drinks [23]. Secondly, variations in associations of different screen-based activities with health may be explained by variations in measurement error, as most evidence on health effects of sedentary behaviour is based on self-report [6]. For example, TV viewing may be easier to recall than other screen-based sedentary activities because TV programs have a known duration. Future research is needed to confirm the observed body movement differences between screen-based and non-screen-based sedentary activities in a real-life setting. Based on the present study we are not able to make any inference about the potential health consequences of these slight differences in body movement. However, Morishima et al. [24] found that intermittent fidgeting counteracted the adverse effects of three hours of prolonged sedentary behaviour on leg endothelial function in healthy young adults (aged 26 years on average). Future studies are needed to examine the long-term health implications and the extent to which slight body movement differences may prevent potential adverse health effects of prolonged sedentary behaviour. 

We used the term ‘screen-based activities’ to group common sedentary activities requiring a screen. However, it was not the purpose of the present study to test whether a specific sedentary activity would elicit less body movement when using a screen. This would have required a different set-up in which participants complete the same activity using a screen or not (e.g., reading a book versus an e-reader).

The results of the present study provide information on the measurement of sedentary behaviour. Accelerometers are commonly used to measure sedentary time, but a limitation is that no contextual information is provided (e.g., TV viewing, sedentary gaming). Time spent in specific sedentary activities is therefore generally assessed by self-report. However, a recent review concluded that no sedentary behaviour questionnaire exists with both an acceptable validity and reliability [25]. The present study shows that it may be possible to distinguish between categories of sedentary activities (e.g., screen-based from non-screen-based sedentary activities by combining data of a thigh-worn accelerometer and heart rate data or distinguishing sedentary activities with arm-movement from sedentary activities without arm-movement by combining data of thigh and wrist-worn accelerometers). Future research should investigate differentiation algorithms to distinguish different sedentary activities.

Hip-worn accelerometers could not distinguish various sedentary activities, as median counts and P75 were mostly zero and differences between sedentary activities and standing were very small. Nevertheless, some small significant differences in hip-counts between the sedentary activities were observed, which can be explained by differences in P90 or higher (data not shown). As data were synchronized with video recordings, we ensured that counts belonging to the highest percentiles truly represented movement during sedentary behaviour.

Wrist-worn accelerometers receive increased attention for obtaining estimates of sedentary behaviour and physical activity [11,12,14], since this position may increase wear compliance [26]. Previous studies have examined both the dominant [12] and the non-dominant hand [11,26]. We showed that counts differed significantly between the dominant and non-dominant hand during sedentary activities. This needs to be taken into account when comparing studies using different positions. We found that counts during sedentary activities were more stable for the non-dominant hand, making it possibly more appropriate for establishing sedentary cut-points, while data from the dominant hand could distinguish sedentary activities requiring arm movement from those requiring no arm-movement. Differences between standing and sedentary activities requiring arm-movement were small, especially for the dominant hand. Differentiating sedentary behaviour from standing is therefore problematic with wrist-mounted as well as hip-mounted accelerometers.

Differences in muscle activity of weight-bearing leg muscles during standing and sedentary activities were small (range: 1.0–3.8% of submaximal muscle activity). Since one explanation for the adverse health effects of sedentary behaviour is a lack of muscle activity of weight-bearing muscles [15,16], the small difference in muscle activity of weight-bearing leg muscles suggests that replacing sitting with standing may be insufficient for clinically relevant benefits. Nonetheless, heart rate was significantly higher during standing versus sedentary activities which may be due to more muscle activity in other muscle groups, e.g., trunk and back muscles to keep the spine erect during standing. Sullivan et al. [27] showed that muscle activity of lumbo-pelvic postural stabilizing muscles was lower in adults during slump sitting and sway standing than during erect sitting and standing, respectively. However, muscle activity during sitting and standing was not compared in that study. Future studies are needed to examine which amount of muscle activity is relevant for health benefits.

The results of the present study provide novel and important knowledge relevant for public health and sedentary behaviour epidemiology. In both children and adolescents, screen-based sedentary activities involved less body movement than non-screen-based sedentary activities. This may be one explanation for why the evidence for adverse health effects is stronger for TV viewing time than for total sedentary time [6]. Nevertheless, future research should examine the potential health effects of the observed differences in body movement between screen-based versus non-screen based and standing versus sedentary activities.

The strengths of this study include the controlled laboratory design; use of video recordings to check compliance to the protocol; use of multiple measurement devices; and the 10-min measurement period for each activity, providing an indication of variation over time. By including children and adolescents, we were able to compare differences in body movement between age groups. Another strength is the variation of common sedentary activities of today’s young people, including ‘media multitasking’ [4]. Finally, we focused our interpretation of differences in body movement on effect sizes and consistency of findings besides statistical significance. One limitation is that we did not measure energy expenditure. However, accelerometer counts and heart rate can validly predict energy expenditure [28,29]. Another limitation is that for logistical reasons, muscle activity was measured in a subgroup of participants. Finally, as with all laboratory research, the lab setting and wearing of the monitors may have influenced the behaviour of our participants.

## 5. Conclusions

Both in children and adolescents, body movement was greater during non-screen-based sedentary activities and standing than during screen-based sedentary activities and lying down. Future studies are needed to confirm these differences in body movement in a real-life setting and should examine whether they are sufficient to impact health on the long term. Moreover, future studies on the measurement of sedentary behaviour are needed to investigate whether the slight body movement differences between sedentary activities are variable enough to recognise specific types of sedentary activities by wearable monitors.

## Figures and Tables

**Table 1:** Participants’ characteristics (mean ± SD).

Characteristics	Children (*n* = 53)	Muscle Activity Sample Children (*n* = 25)	Adolescents (*n* = 37)	Muscle Activity Sample Adolescents (*n* = 13)
Age (year)	12.0 ± 0.8	12.0 ± 0.9	17.4 ± 0.9	17.5 ± 1.1
% Boys	57	56	51	54
Height (cm)	155.8 ± 8.2	155.0 ± 7.1	176.2 ± 8.0	175.7 ± 8.6
Weight (kg)	45.3 ± 8.6	44.4 ± 6.4	65.6 ± 8.5	67.7 ± 10.0
BMI (kg/m^2^)	18.6 ± 2.9	18.4 ± 2.1	21.1 ± 2.2	21.9 ± 2.7
% Overweight/obese	17	12	5	15

BMI, body mass index; SD, standard deviation.

**Table 2:** Description of the nine activities and order in which activities were performed (random start activity).

Type	Activity	Description of Activity	Order
**Lying down**	Lying down	Lie on a mat on back or left or right side. Instructed to lie calm.	1
**Sitting**	Using a tablet	Sit in a lounge chair and use a tablet (surfing internet, play a game etc.).	2
	Watching movies	Sit in a lounge chair and watch movies (selection of age-appropriate educational movies) on a laptop which was placed on a low table.	3
	Drawing/writing	Sit in a chair at a desk and use felt pens and paper to make a drawing, comic strip, write a story or write down as many words related to sitting as possible. Instructed to keep drawing or writing.	4
	Computer gaming	Sit in a chair at a desk and play a sedentary computer game on a laptop (using a keyboard).	5
	Just sitting	Sit in a chair and wait for the following activity, no additional imposed activity (using smartphones, etc. was not allowed).	7
	Media multitasking	Sit in a lounge chair and watch a movie (selection of age-appropriate educational movies) on a laptop which was placed on a low table, while using a tablet (free choice, internet available).	8
**Standing**	Standing	Standing straight within a square of 0.75 m^2^ that was taped on the floor. Instructed to stay within this square allowing small movements, but no jumping.	9
**Moving**	Dancing	Dance along with dance movies from the active video game “Just Dance”.	6

**Table 3:** Medians (25th and 75th percentiles) of different body movement variables for different activities in children.

Body Movement Variables	Lying Down	Watching Movies	Computer Gaming	Tablet Use	Media Multitasking	Just Sitting	Drawing	Standing	Dancing
*Accelerometer* *									
Hip *X*-axis	0 (0; 0)	0 (0; 0)	0 (0; 0)	0 (0; 0)	0 (0; 0)	0 (0; 2)	0 (0; 26)	17 (0; 65)	257 (111; 446)
Hip *Y*-axis	0 (0; 0)	0 (0; 0)	0 (0; 0)	0 (0; 0)	0 (0; 0)	0 (0; 0)	0 (0; 0)	0 (0; 6)	119 (27; 462)
Hip *Z*-axis	0 (0; 0)	0 (0; 0)	0 (0; 4)	0 (0; 0)	0 (0; 0)	0 (0; 9)	8 (0; 45)	26 (0; 97)	271 (126; 486)
Thigh *X*-axis	1 (0; 18)	1 (0; 51)	2 (0; 70)	2 (0; 55)	2 (0; 53)	31 (1; 143)	41 (2; 114)	225 (68; 481)	1807 (735; 3815)
Thigh *Y*-axis	1 (0; 38)	1 (0; 91)	2 (1; 108)	3 (1; 77)	2 (1; 77)	48 (1; 212)	55 (2; 179)	458 (155; 868)	3020 (1459; 5268)
Thigh *Z*-axis	1 (0; 26)	1 (0; 52)	2 (0; 62)	2 (0; 58)	2 (0; 55)	28 (1; 131)	30 (1; 92)	324 (113; 619)	1950 (951; 3390)
Wrist dominant *X*-axis	8 (0; 270)	0 (0; 139)	24 (0; 101)	160 (18; 397)	156 (26; 409)	123 (0; 351)	185 (35; 467)	235 (54; 541)	2150 (1218; 2899)
Wrist dominant *Y*-axis	0 (0; 147)	0 (0; 44)	0 (0; 24)	47 (0; 150)	53 (3; 161)	45 (0; 220)	137 (25; 307)	214 (26; 606)	2455 (1478; 3289)
Wrist dominant *Z*-axis	6 (0; 263)	0 (0; 144)	0 (0; 11)	141 (17; 332)	143 (12; 350)	123 (0; 380)	282 (106; 547)	266 (57; 573)	1818 (1095; 2542)
Wrist non-dominant *X*-axis	3 (0; 247)	5 (0; 185)	10 (0; 109)	14 (0; 176)	17 (0; 147)	112 (0; 366)	176(26; 409)	259 (61; 552)	1813 (1032; 2589)
Wrist non-dominant *Y*-axis	0 (0; 144)	0 (0; 37)	0 (0; 40)	0 (0; 61)	0 (0; 54)	28 (0; 189)	50 (0; 205)	207 (31; 587)	2174 (1251; 3098)
Wrist non-dominant *Z*-axis	2 (0; 273)	0 (0; 110)	0 (0; 29)	53 (0; 229)	62 (0; 235)	85 (0; 320)	86 (0; 308)	288 (62; 575)	1616 (914; 2338)
*Heart rate* ^§^	76 (70; 84)	78 (69; 86)	80 (72; 87)	79 (73; 86)	81 (74; 90)	84 (76; 93)	84 (77; 91)	96 (88; 104)	110 (98; 123)
*Muscle activity* ^#^									
Vastus lateralis	1.2 (0.9; 2.0)	1.4 (0.9; 2.7)	1.3 (0.9; 2.5)	1.0 (0.6; 1.9)	0.9 (0.8; 1.4)	1.4 (0.8; 2.5)	1.2 (0.7; 1.9)	2.4 (1.3; 4.6)	7.8 (4.3; 13.2)
Rectus femoris	1.9 (1.4; 4.3)	1.6 (1.3; 2.8)	2.8 (1.7; 4.6)	1.7 (1.3; 3.8)	2.0 (1.4; 4.2)	2.0 (1.4; 5.7)	1.8 (1.3; 3.0)	3.1 (1.8; 5.7)	9.7 (5.4; 16.7)
Gastrocnemius medialis	1.3 (0.9; 3.1)	1.2 (0.9; 2.4)	1.7 (1.1; 3.8)	1.3 (0.9; 1.9)	1.7 (1.1; 4.1)	1.3 (0.8; 1.9)	1.3 (1.0; 1.8)	4.4 (2.3; 7.2)	7.5 (4.7; 12.2)

* counts/15-s; ^§^ beats/minute; ^#^ percentage of submaximal muscle activity.

**Table 4:** Medians (25th and 75th percentiles) of different body movement variables for different activities in adolescents.

Body Movement Variables	Lying Down	Watching Movies	Computer Gaming	Tablet Use	Media Multitasking	Just Sitting	Drawing	Standing	Dancing
*Accelerometer* *									
Hip *X*-axis	0 (0; 0)	0 (0; 0)	0 (0; 0)	0 (0; 0)	0 (0; 0)	0 (0; 0)	0 (0; 0)	0 (0; 8)	333 (154; 539)
Hip *Y*-axis	0 (0; 0)	0 (0; 0)	0 (0; 0)	0 (0; 0)	0 (0; 0)	0 (0; 0)	0 (0; 0)	0 (0; 0)	264 (48; 780)
Hip *Z*-axis	0 (0; 0)	0 (0; 0)	0 (0; 0)	0 (0; 0)	0 (0; 0)	0 (0; 0)	0 (0; 0)	0 (0; 13)	323 (166; 536)
Thigh *X*-axis	0 (0; 1)	0 (0; 1)	1 (0; 3)	1 (0; 3)	1 (0; 9)	1 (0; 25)	1 (0; 27)	43 (1; 147)	2633 (1205; 4746)
Thigh *Y*-axis	0 (0; 1)	1 (0; 2)	1 (0; 4)	1 (0; 3)	1 (0; 12)	1 (0; 32)	1 (0; 40)	100 (2; 281)	3582 (1874; 5973)
Thigh *Z*-axis	0 (0; 1)	1 (0; 2)	1 (0; 3)	1 (0; 2)	1 (0; 10)	1 (0; 22)	1 (0; 23)	74 (2; 200)	2413 (1250; 3919)
Wrist dominant *X*-axis	0 (0; 0)	0 (0; 22)	0 (0; 31)	70 (0; 261)	94 (3; 268)	0 (0; 116)	136 (18; 373)	36 (0; 202)	2605 (1636; 3499)
Wrist dominant *Y*-axis	0 (0; 0)	0 (0; 0)	0 (0; 7)	18 (0; 83)	18 (0; 85)	0 (0; 57)	64 (9; 175)	10 (0; 203)	2838 (1948; 3666)
Wrist dominant *Z*-axis	0 (0; 0)	0 (0; 5)	0 (0; 0)	24 (0; 221)	50 (0; 220)	0 (0; 173)	241 (84; 515)	24 (0; 226)	2135 (1436; 2925)
Wrist non-dominant *X*-axis	0 (0; 3)	0 (0; 42)	0 (0; 47)	0 (0; 39)	0 (0; 41)	0 (0; 177)	79 (0; 268)	64 (0; 332)	2244 (1434; 3034)
Wrist non-dominant *Y*-axis	0 (0; 0)	0 (0; 0)	0 (0; 0)	0 (0; 4)	0 (0; 2)	0 (0; 71)	11 (0; 91)	28 (0; 321)	2640 (1715; 3372)
Wrist non-dominant *Z*-axis	0 (0; 0)	0 (0; 15)	0 (0; 5)	0 (0; 103)	0 (0; 67)	0 (0; 171)	14 (0; 198)	64 (0; 314)	1919 (1226; 2570)
*Heart rate* ^§^	69 (62; 75)	69 (62; 79)	73 (67; 81)	71 (63; 81)	75 (66; 82)	82 (72; 90)	77 (69; 86)	91 (81; 101)	114 (99; 132)
*Muscle activity* ^#^									
Vastus lateralis	0.9 (0.6; 1.8)	1.1 (0.6; 1.5)	1.2 (0.7; 2.0)	1.0 (0.7; 2.1)	1.2 (0.9; 3.1)	1.5 (1.0; 3.7)	0.9 (0.6; 1.4)	3.3 (1.2; 6.0)	13.3 (8.7; 20.2)
Rectus femoris	1.9 (1.0; 2.8)	1.9 (0.9; 3.7)	3.9 (1.1; 5.8)	2.1 (0.9; 3.4)	2.7 (1.0; 4.8)	3.1 (1.4; 6.1)	2.1 (0.9; 4.4)	4.1 (1.9; 5.6)	14.5 (6.9; 29.0)
Gastrocnemius medialis	0.6 (0.5; 0.9)	0.9 (0.7; 1.4)	1.1 (0.7; 1.8)	0.9 (0.7; 1.5)	1.0 (0.6; 1.5)	0.9 (0.6; 1.2)	0.8 (0.7; 1.5)	3.2 (1.9; 5.6)	11.2 (6.6; 23.0)

* counts/15-s; ^§^ beats/minute; ^#^ percentage of submaximal muscle activity.

## Appendix A

### Appendix A.1. Results of the GEE Analyses for All Indicators of Body Movement

**Table A1:** Comparison of hip-based accelerometer counts between different activities in children and adolescents.

Activities	Children			Adolescents		
	Hip *X*-axis (counts/15 s)	Hip *Y*-axis (counts/15 s)	Hip *Z*-axis (counts/15 s)	Hip *X*-axis (counts/15 s)	Hip *Y*-axis (counts/15 s)	Hip *Z*-axis (counts/15 s)
*Compared to watching movies*						
lying down	1.6 (0.96; 2.6)	1.1 (0.6; 1.9)	0.8 (0.5; 1.2)	0.5 (0.2; 1.8)	0.8 (0.4; 1.6)	0.7 (0.2; 2.1)
computer gaming	0.9 (0.5; 1.5)	**0.4 (0.3; 0.7)**	0.7 (0.4; 1.2)	0.7 (0.4; 1.2)	0.3 (0.1; 0.7)	0.8 (0.4; 1.7)
tablet use	**0.4 (0.2; 0.8)**	0.7 (0.4; 1.1)	**0.4 (0.2; 0.8)**	0.5 (0.2; 1.3)	1.2 (0.5; 2.9)	0.6 (0.3; 1.4)
media multitasking	1.0 (0.6; 1.7)	0.8 (0.5; 1.5)	0.8 (0.4; 1.6)	0.6 (0.3; 1.3)	0.8 (0.4; 1.3)	0.7 (0.3; 1.5)
just sitting	**1.7 (1.1; 2.8)**	1.4 (0.9; 2.4)	1.5 (0.8; 2.7)	1.6 (0.8; 3.0)	1.8 (0.9; 3.6)	1.9 (0.9; 3.9)
drawing	**2.3 (1.4; 3.8)**	1.1 (0.5; 2.3)	**2.0 (1.2; 3.4)**	1.2 (0.6; 2.5)	**0.3 (0.1; 0.7)**	1.3 (0.6; 2.7)
standing	**5.4 (3.2; 9.1)**	**2.7 (1.1; 7.0)**	**3.8 (2.1; 6.9)**	**4.3 (2.3; 7.9)**	0.8 (0.4; 1.6)	**4.6 (2.7; 7.7)**
dancing	**31.4 (19.1; 51.5)**	**41.7 (23.9; 72.6)**	**16.9 (9.3; 30.9)**	**143.6 (86.1; 239.6)**	**317.0 (179.8; 558.9)**	**110.7 (62.6; 195.8)**
*Compared to lying down*						
computer gaming	**0.5 (0.3; 0.9)**	1.0 (0.5; 1.8)	0.9 (0.7; 1.3)	1.2 (0.3; 4.4)	0.7 (0.2; 2.2)	1.2 (0.4; 3.8)
tablet use	**0.3 (0.2; 0.4)**	0.7 (0.4; 1.0)	**0.5 (0.3; 0.8)**	1.0 (0.6; 6.1)	1.4 (0.4; 4.9)	0.9 (0.3; 3.3)
media multitasking	**0.6 (0.4; 1.0)**	0.8 (0.4; 1.3)	1.1 (0.6; 1.8)	1.2 (0.3; 4.3)	0.9 (0.4; 1.9)	1.1 (0.3; 3.4)
just sitting	1.1 (0.7; 1.7)	1.3 (0.9; 2.0)	**2.0 (1.2; 3.3)**	2.9 (0.8; 10.2)	2.1 (0.8; 5.4)	2.9 (1.0; 8.8)
drawing	**1.5 (1.1; 2.1)**	1.0 (0.5; 1.8)	**2.7 (1.9; 3.8)**	2.2 (0.6; 8.2)	1.3 (0.7; 2.8)	2.0 (0.6; 6.5)
standing	**3.4 (2.3; 5.1)**	**2.5 (1.1; 5.8)**	**5.1 (3.4; 7.6)**	**7.9 (2.8; 21.9)**	1.0 (0.5; 2.0)	**7.0 (2.5; 20.5)**
dancing	**19.9 (14.0; 28.4)**	**38.4 (23.5; 62.7)**	**22.5 (15.1; 33.6)**	**265.8 (88.5; 798.7)**	**377.8 (178.5; 799.9)**	**169.7 (63.3; 454.7)**
*Compared to computer gaming*						
tablet use	**0.5 (0.3; 0.9)**	1.6 (1.0; 2.8)	**0.5 (0.3; 0.9)**	0.8 (0.4; 1.6)	2.0 (0.6; 6.8)	0.8 (0.4; 1.6)
media multitasking	1.2 (0.7; 2.0)	**1.9 (1.1; 3.2)**	1.1 (0.7; 1.9)	1.0 (0.6; 1.7)	1.3 (0.5; 3.1)	0.9 (0.4; 1.8)
just sitting	**2.0 (1.3; 3.1)**	**3.3 (2.1; 5.1)**	**2.2 (1.5; 3.2)**	**2.4 (1.4; 4.1)**	**3.0 (1.2; 7.5)**	**2.4 (1.3; 4.4)**
drawing	**2.7 (1.8; 4.1)**	**2.5 (1.3; 4.8)**	**2.9 (2.1; 4.0)**	1.8 (0.9; 3.5)	0.5 (0.2; 1.3)	1.6 (0.8; 3.1)
standing	**6.3 (4.0; 9.8)**	**6.3 (2.5; 15.6)**	**5.5 (3.8; 7.7)**	**6.4 (3.2; 12.9)**	1.4 (0.5; 4.4)	**5.7 (3.1; 10.6)**
dancing	**36.4 (24.4; 54.4)**	**95.8 (60.1; 152.7)**	**24.1 (16.9; 34.4)**	**216.1 (130.7; 357.2)**	**533.5 (224.3; 1268.7)**	**138.3 (91.0; 210.4)**
*Compared to tablet use*						
media multitasking	**2.4 (1.5; 3.9)**	1.2 (0.7; 1.9)	**2.2 (1.3; 3.6)**	1.3 (0.5; 3.2)	0.6 (0.2; 1.8)	1.1 (0.5; 2.6)
just sitting	**4.0 (2.4; 6.6)**	**2.0 (1.3; 3.1)**	**4.1 (2.5; 6.6)**	**3.1 (1.3; 7.4)**	1.5 (0.6; 3.8)	**3.1 (1.3; 7.2)**
drawing	**5.4 (3.3; 9.0)**	1.5 (0.8; 2.8)	**5.4 (3.3; 8.7)**	2.3 (0.8; 6.4)	**0.3 (0.08; 0.9)**	2.1 (1.0; 4.5)
standing			**12.6 (8.4; 18.8)**	**3.8 (1.8; 8.2)**	0.7 (0.2; 2.4)	**7.4 (3.4; 16.1)**
dancing	**73.1 (47.4; 112.8)**	**58.2 (36.9; 91.8)**	**45.5 (27.3; 75.9)**	**279.3 (108.3; 720.5)**	**268.7 (104.9; 688.2)**	**179.6 (89.5; 360.5)**
*Compared to media multitasking*						
just sitting	**1.7 (1.1; 2.6)**	**1.7 (1.1; 2.6)**	**1.9 (1.3; 2.8)**	**2.4 (1.3; 4.5)**	**2.4 (1.3; 4.3)**	**2.8 (1.4; 5.6)**
drawing	**2.9 (1.6; 3.3)**	1.3 (0.7; 2.4)	**2.5 (1.7; 3.7)**	**1.8 (1.2; 2.9)**	**0.4 (0.2; 0.8)**	1.9 (1.0; 3.6)
standing	**5.3 (3.6; 7.6)**	**3.3 (1.4; 7.4)**	**4.8 (3.3; 6.9)**	**6.6 (3.3; 12.9)**	1.1 (0.5; 2.4)	**6.7 (3.3; 13.3)**
dancing	**30.7 (21.7; 43.5)**	**50.0 (32.7; 76.6)**	**21.0 (14.3; 31.0)**	**221.2 (125.4; 390.2)**	**419.9 (268.2; 657.3)**	**161.1 (87.3; 297.2)**
*Compared to just sitting*						
drawing	1.4 (0.9; 2.1)	0.8 (0.4; 1.4)	1.3 (0.9; 1.8)	0.8 (0.4; 1.5)	**0.2 (0.08; 0.4)**	0.7 (0.3; 1.3)
Standing	**3.2 (2.1; 4.7)**	1.9 (0.8; 4.3)	**2.5 (1.8; 3.5)**	**2.7 (1.4; 5.2)**	0.5 (0.2; 1.2)	**2.4 (1.3; 4.4)**
dancing	**18.3 (12.3; 27.3)**	**29.3 (20.8; 41.3)**	**11.2 (7.9; 15.7)**	**91.2 (58.8; 141.4)**	**177.4 (101.2; 310.7)**	**57.9 (37.8; 88.8)**
*Compared to drawing*						
standing	**2.3 (1.7; 3.1)**	1.0 (0.5; 1.9)	**1.9 (1.5; 2.5)**	**3.6 (1.8; 7.2)**	**2.7 (1.0; 6.8)**	**3.5 (1.7; 7.3)**
dancing	**13.4 (10.5; 17.2)**	**38.9 (22.5; 67.1)**	**8.5 (6.5; 11.0)**	**121.2 (61.5; 238.8)**	**1020.5 (487.8; 2134.7)**	**85.6 (46.2; 158.6)**
*Compared to standing*						
dancing	**5.8 (4.5; 7.6)**	**15.3 (6.9; 34.0)**	**24.2 (14.8; 39.4)**	**33.7 (20.7; 55.1)**	**379.6 (169.8; 848.6)**	**24.2 (14.8; 39.4)**

Data in bold indicate statistical significant differences (*p* < 0.05). Activities are sorted from smallest to greatest body movement, based on the general trend for all different devices. s, seconds.

**Table A2:** Comparison of thigh-based accelerometer counts between different activities in children and adolescents.

Activities	Children			Adolescents		
	Thigh *X*-axis (counts/15 s)	Thigh *Y*-axis (counts/15 s)	Thigh *Z*-axis (counts/15 s)	Thigh *X*-axis (counts/15 s)	Thigh *Y*-axis (counts/15 s)	Thigh *Z*-axis (counts/15 s)
*Compared to watching movies*						
lying down	0.8 (0.6; 1.1)	1.0 (0.7; 1.5)	1.2 (0.9; 1.7)	0.7 (0.3; 1.6)	0.8 (0.4; 1.7)	0.9 (0.4; 2.1)
computer gaming	**1.6 (1.1; 2.6)**	1.6 (1.0; 2.3)	2.0 (1.0; 4.1)	**2.3 (1.1; 5.1)**	**3.5 (1.3; 9.4)**	**2.4 (1.0; 5.5)**
tablet use	1.2 (0.9; 1.5)	1.1 (0.8; 1.4)	1.5 (1.1; 2.2)	1.2 (0.8; 1.8)	1.5 (0.9; 2.6)	1.3 (0.8; 2.0)
media multitasking	1.0 (0.8; 1.4)	1.1 (0.8; 1.5)	1.1 (0.9; 1.4)	1.2 (0.8; 1.8)	1.6 (0.8; 3.2)	1.3 (0.8; 2.3)
just sitting	**2.5 (1.8; 3.5)**	**2.2 (1.7; 2.9)**	**2.6 (1.9; 3.7)**	**3.0 (1.7; 5.4)**	**2.5 (1.4; 4.4)**	**3.5 (1.7; 7.1)**
drawing	**2.0 (1.6; 2.7)**	**1.9 (1.5; 2.5)**	**1.8 (1.4; 2.3)**	**3.1 (1.4; 6.6)**	**4.7 (1.6; 14.0)**	**3.2 (1.3; 7.9)**
standing	**8.1 (6.2; 10.6)**	**9.1 (7.1; 11.7)**	**10.3 (8.1; 13.3)**	**8.4 (5.3; 13.3)**	**11.5 (7.8; 16.8)**	**11.6 (7.0; 19.2)**
dancing	**58.2 (44.0; 76.9)**	**52.8 (40.1; 69.5)**	**55.8 (42.2; 73.7)**	**248.0 (158.6; 387.7)**	**225.5 (147.8; 343.8)**	**212.8 (127.8; 354.2)**
*Compared to lying down*						
computer gaming	**2.0 (1.2; 3.3)**	1.5 (1.0; 2.3)	1.7 (0.8; 3.4)	**3.3 (1.2; 8,9)**	**4.5 (1.4; 14.1)**	2.7 (1.0; 7.7)
tablet use	**1.4 (1.1; 2.0)**	1.1 (0.8; 1.5)	1.3 (0.9; 1.8)	1.8 (0.8; 4.0)	1.9 (0.8; 4.6)	1.5 (0.6; 3.4)
media multitasking	1.3 (0.9; 1.7)	1.0 (0.7; 1.5)	0.9 (0.7; 1.2)	1.7 (0.8; 3.9)	2.0 (0.8; 5.40	1.5 (0.7; 3.6)
just sitting	**3.1 (2.2; 4.2)**	**2.2 (1.6; 3.0)**	**2.2 (1.6; 3.0)**	**4.3 (2.0; 9.1)**	**3.3 (1.6; 6.6)**	**4.0 (1.7; 9.3)**
drawing	**2.5 (1.8; 3.5)**	**1.9 (1.3; 2.6)**	**1.5 (1.0; 2.1)**	**4.4 (1.6; 11.9)**	**6.1 (1.8; 21.0)**	**3.7 (1.3; 10.8)**
standing	**9.9 (7.2; 13.6)**	**8.9 (6.5; 12.2)**	**8.6 (6.3; 11.7)**	**12.1 (5.3; 27.2)**	**14.8 (6.9; 31.9)**	**13.4 (5.9; 30.4)**
dancing	**71.0 (52.1; 96.6)**	**51.4 (38.0; 69.5)**	**46.4 (34.6; 62.3)**	**355.5 (170.2; 742.6)**	**290.8 (144.1; 587.1)**	**246.4 (115.5; 525.5)**
*Compared to computer gaming*						
tablet use	0.7 (0.5; 1.1)	0.7 (0.5; 1.0)	0.8 (0.4; 1.5)	0.5 (0.3; 1.0)	0.4 (0.2; 1.0)	0.5 (0.3; 1.0)
media multitasking	0.6 (0.4; 1.0)	0.7 (0.4; 1.1)	0.6 (0.3; 1.1)	**0.5 (0.3; 0.9)**	**0.5 (0.3; 0.7)**	**0.6 (0.3; 1.0)**
just sitting	1.5 (0.9; 2.6)	1.4 (0.9; 2.3)	1.3 (0.6; 2.9)	1.3 (0.7; 2.5)	0.7 (0.3; 1.7)	1.5 (0.7; 3.2)
drawing	1.2 (0.8; 2.0)	1.2 (0.8; 1.9)	0.9 (0.4; 1.8)	1.3 (0.8; 2.2)	**1.4 (1.0; 1.8)**	1.4 (0.7; 2.9)
standing	**4.9 (3.0; 8.0)**	**5.9 (3.8; 9.0)**	**5.2 (2.5; 10.7)**	**3.6 (1.6; 8.0)**	**3.3 (1.2; 8.8)**	**4.9 (2.2; 11.1)**
dancing	**35.3 (21.6; 57.7)**	**34.0 (21.8; 53.1)**	**28.0 (13.3; 58.8)**	**106.3 (54.3; 208.1)**	**64.8 (26.2; 160.2)**	**90.3 (46.3; 176.1)**
*Compared to tablet use*						
media multitasking	0.9 (0.7; 1.2)	1.0 (0.7; 1.3)	0.7 (0.5; 1.0)	1.0 (0.7; 1.3)	1.0 (0.6; 1.9)	1.0 (0.7; 1.5)
just sitting	**2.1 (1.6; 2.8)**	**2.0 (1.5; 2.6)**	**1.7 (1.2; 2.5)**	**2.4 (1.5; 3.8)**	1.7 (1.0; 2.9)	**2.7 (1.5; 4.8)**
drawing	**1.7 (1.3; 2.3)**	**1.7 (1.3; 2.3)**	1.2 (0.8; 1.7)	**2.5 (1.3; 4.8)**	**3.1 (1.2; 8.2)**	**2.5 (1.2; 5.4)**
standing	**6.9 (5.4; 8.8)**	**8.3 (6.3; 10.9)**	**6.8 (5.0; 9.2)**	**6.8 (4.4; 10.5)**	**7.6 (4.6; 12.40**	**9.0 (6.2; 13.3)**
dancing	**49.4 (36.1; 67.8)**	**48.0 (35.1; 65.4)**	**36.7 (25.6; 52.7)**	**199.8 (140.3; 284.4)**	**149.2 (93.6; 237.8)**	**165.9 (118.5; 232.4)**
*Compared to media multitasking*						
just sitting	**2.4 (1.8; 3.4)**	**2.1 (1.5; 2.9)**	**2.4 (1.7; 3.3)**	**2.5 (1.6; 3.9)**	1.6 (0.9; 3.0)	**2.6 (1.5; 4.6)**
drawing	**2.0 (1.5; 2.7)**	**1.8 (1.2; 2.7)**	**1.6 (1.2; 2.2)**	**2.6 (1.4; 4.9)**	**3.0 (1.8; 4.9)**	**2.4 (1.2; 4.7)**
standing	**7.8 (5.9; 10.3)**	**8.5 (6.0; 12.0)**	**9.3 (7.2; 12.0)**	**7.1 (4.5; 11.2)**	**7.3 (3.6; 14.6)**	**8.7 (5.4; 14.1)**
dancing	**56.0 (43.3; 72.5)**	**49.3 (35.4; 68.8)**	**50.2 (39.7; 63.5)**	**208.1 (149.0; 290.7)**	**142.8 (74.8; 272.6)**	**159.9 (108.5; 235.7)**
*Compared to just sitting*						
drawing	0.8 (0.6; 1.1)	0.9 (0.7; 1.2)	**0.7 (0.5; 0.9)**	1.0 (0.6; 1.8)	1.9 (0.8; 4.6)	0.9 (0.5; 1.6)
Standing	**3.2 (2.4; 4.3)**	**4.1 (3.1; 5.5)**	**3.9 (2.9; 5.3)**	**2.8 (1.7; 4.5)**	**4.5 (2.9; 7.2)**	**3.3 (2.0; 5.7)**
dancing	**23.0 (16.7; 31.9)**	**23.8 (17.8; 31.9)**	**21.1 (15.3; 29.0)**	**82.4 (56.8; 119.7)**	**89.2 (63.1; 126.1)**	**61.3 (38.0; 98.8)**
*Compared to drawing*						
standing	**4.0 (3.0; 5.3)**	**4.7 (3.6; 6.2)**	**5.9 (4.4; 7.8)**	**2.8 (1.3; 5.8)**	2.4 (0.8; 7.0)	**3.6 (1.6; 7.9)**
dancing	**28.4 (21.5; 37.4)**	**27.4 (20.9; 36.1)**	**31.7 (23.9; 42.0)**	**81.0 (41.3; 159.2)**	**47.6 (17.4; 130.4)**	**66.2 (31.3; 139.7)**
*Compared to standing*						
dancing	**7.2 (5.6; 9.3)**	**5.8 (4.7; 7.2)**	**5.4 (4.4; 6.6)**	**29.5 (18.8; 46.2)**	**19.7 (13.0; 29.6)**	**18.3 (12.1; 27.7)**

Data in bold indicate statistical significant differences (*p* < 0.05). Activities are sorted from smallest to greatest body movement, based on the general trend for all different devices. s, seconds.

**Table A3:** Comparisons of wrist-based accelerometer counts between different activities in children.

Activities	Wrist Dominant *X*-axis (counts/15 s)	Wrist Dominant *Y*-axis (counts/15 s)	Wrist Dominant *Z*-axis (counts/15 s)	Wrist Non-Dominant *X*-axis (counts/15 s)	Wrist Non-Dominant *Y*-axis (counts/15 s)	Wrist Non-Dominant *Z*-axis (counts/15 s)
*Compared to watching movies*						
lying down	**1.5 (1.2; 2.0)**	**1.7 (1.3; 2.2)**	**1.8 (1.3; 2.4)**	**1.4 (1.0; 1.8)**	**1.9 (1.4; 2.5)**	**1.9 (1.4; 2.5)**
computer gaming	**0.7 (0.6; 0.9)**	**0.6 (0.5; 0.8)**	**0.4 (0.3; 0.6)**	**0.7 (0.6; 0.9)**	0.9 (0.7; 1,2)	**0.6 (0.5; 0.8)**
tablet use	**2.1 (1.7; 2.6)**	**1.6 (1.3; 2.1)**	**2.3 (1.8; 3.0)**	1.0 (0.8; 1.3)	1.0 (0.8; 1.3)	**1.5 (1.2; 2.0)**
media multitasking	**2.2 (1.8; 2.8)**	**1.7 (1.4; 2.2)**	**2.3 (1.8; 3.0)**	1.0 (0.8; 1.3)	1.1 (0.8; 1.4)	**1.5 (1.2; 2.0)**
just sitting	**2.1 (1.7; 2.6)**	**2.3 (1.9; 2.9)**	**2.6 (2.0; 3.2)**	**1.9 (1.5; 2.4)**	**2.4 (1.9; 3.0)**	**2.2 (1.7; 2.8)**
drawing	**2.5 (2.0; 3.1)**	**3.1 (2.5; 3.9)**	**3.8 (3.0; 4.9)**	**2.1 (1.7; 2.6)**	**2.3 (1.9; 2.9)**	**2.1 (1.7; 2.6)**
standing	**3.2 (2.5; 4.1)**	**6.5 (5.1; 8.2)**	**4.1 (3.1; 5.3)**	**1.4 (1.0; 1.8)**	**7.4 (5.8; 9.6)**	**4.1 (3.2; 5.2)**
dancing	**17.6 (13.7; 22.7)**	**36.5 (28.6; 46.7)**	**19.5 (15.3; 24.9)**	**14.6 (11.5; 18.5)**	**38.9 (30.6; 49.6)**	**17.4 (13.7; 21.9)**
*Compared to lying down*						
computer gaming	**0.5 (0.4; 0.6)**	**0.4 (0.3; 0.5)**	**0.2 (0.2 0.3)**	**0.5 (0.4; 0.7)**	**0.5 (0.4; 0.7)**	**0.3 (0.3; 0.5)**
tablet use	**1.4 (1.1; 1.7)**	1.0 (0.8; 1.2)	**1.3 (1.0; 1.6)**	**0.8 (0.6; 1.0)**	**0.5 (0.4; 0.7)**	0.8 (0.7; 1.1)
media multitasking	**1.4 (1.1; 1.8)**	1.0 (0.8; 1.3)	**1.3; 1.0; 1.7)**	0.8 (0.6; 1.0)	**0.6 (0.4; 0.8)**	0.8 (0.7; 1.1)
just sitting	**1.3 (1.1; 1.7)**	**1.4 (1.1; 1.8)**	**1.4 (1.2; 1.8)**	**1.4 (1.1; 1.7)**	**1.3 (1.1; 1.6)**	1.2 (1.0; 1.4)
drawing	**1.6 (1.3; 2.1)**	**1.9 (1.5; 2.4)**	**2.2 (1.7; 2.7)**	**1.5 (1.2; 2.0)**	**1.3 (1.0; 1.6)**	1.1 (0.9; 1.4)
standing	**2.1 (1.6; 2.7)**	**3.9 (2.9; 5.1)**	**2.3 (1.8; 2.9)**	**2.3 (1.8; 3.0)**	**4.0 (3.1; 5.2)**	**2.2 (1.7; 2.8)**
dancing	**11.6 (8.9; 15.0)**	**21.9 (16.6; 28.8)**	**10.9 (8.4; 14.2)**	**10.7 (8.1; 14.2)**	**20.9 (16.0; 27.4)**	**9.4 (7.2; 12.2)**
*Compared to computer gaming*						
tablet use	**2.9 (2.4; 3.6)**	**2.6 (2.0; 3.5)**	**5.4 (4.1; 7.1)**	**1.4 (1.1; 1.8)**	1.1 (0.8; 1.4)	**2.4 (1.8; 3.1)**
media multitasking	**3.0 (2.5; 3.7)**	**2.8 (2.1; 3.6)**	**5.4 (4.3; 6.8)**	**1.4 (1.1; 1.9)**	1.2 (0.8; 1.6)	**2.4 (1.8; 3.1)**
just sitting	**2.9 (2.3; 3.5)**	**3.8 (2.9; 4.8)**	**5.9 (4.6; 7.6)**	**2.7 (2.1; 3.4)**	**2.6 (1.9; 3.4)**	**3.4 (2.6; 4.5)**
drawing	**3.5 (2.9; 4.2)**	**5.0 (3.9; 6.5)**	**8.9 (7.1; 11.3)**	**2.9 (2.5; 3.5)**	**2.5 (2.0; 3.2)**	**3.3 (2.6; 4.1)**
standing	**4.5 (3.6; 5.5)**	**2.7 (1.9; 3.7)**	**9.4 (7.4; 12.1)**	**4.4 (3.6; 5.4)**	**8.0 (6.1; 10.6)**	**6.3 (4.9; 8.1)**
dancing	**24.5 (20.1; 29.7)**	**58.6 (45.5; 75.4)**	**45.3 (35.4; 58.0)**	**20.5 (16.5; 25.6)**	**41.9 (32.2; 54.3)**	**26.8 (20.8; 34.6)**
*Compared to tablet use*						
media multitasking	1.0 (0.9; 1.2)	1.1 (0.9; 1.3)	1.0 (0.8; 1.2)	1.0 (0.8; 1.2)	1.1 (0.8; 1.3)	1.0 (0.9; 1.2)
just sitting	1.0 (0.8; 1.2)	**1.4 (1.2; 1.8)**	1.1 (0.9; 1.3)	**1.9 (1.4; 2.4)**	**2.3 (1.8; 3.1)**	**1.4 (1.2; 1.8)**
drawing	**1.2 (1.0; 1.4)**	**1.9 (1.5; 2.4)**	**1.6 (1.4; 2.0)**	**2.0 (1.7; 2.5)**	**2.3 (1.9; 2.9)**	**1.4 (1.1; 1.6)**
standing	**1.5 (1.2; 1.9)**	**4.0 (3.1; 5.2)**	**1.7 (1.4; 2.2)**	**3.1 (2.4; 3.9)**	**7.3 (5.8; 9.4)**	**2.6 (2.2; 3.2)**
dancing	**8.3 (7.0; 9.9)**	**22.5 (18.2; 27.8)**	**8.3 (6.9; 10.1)**	**14.2 (11.5; 17.5)**	**38.4 (30.8; 47.4)**	**11.3 (9.3; 13.7)**
*Compared to Media multitasking*						
just sitting	0.9 (0.8; 1.1)	**1.4 (1.1; 1.7)**	1.1 (0.9; 1.3)	**1.9 (1.4; 2.5)**	**2.2 (1.7; 2.9)**	**1.4 (1.1; 1.8)**
drawing	1.1 (1.0; 1.3)	**1.8 (1.5; 2.2)**	**1.7 (1.4; 2.0)**	**2.0 (1.6; 2.6)**	**2.2 (1.7; 2.8)**	**1.4 (1.1; 1.6)**
standing	**1.5 (1.2; 1.8)**	**3.7 (2.9; 4.8)**	**1.7 (1.4; 2.1)**	**3.1 (2.4; 3.9)**	**6.9 (5.2; 9.2)**	**2.6 (2.2; 3.1)**
dancing	**8.0 (7.0; 9.2)**	**21.1 (17.9; 24.9)**	**8.4 (7.2; 9.7)**	**14.2 (11.4; 17.7)**	**36.2 (27.4; 47.7)**	**11.2 (9.2; 13.7)**
*Compared to just sitting*						
drawing	**1.2 (1.0; 1.5)**	**1.3 (1.1; 1.6)**	**1.5 (1.3; 1.7)**	1.1 (0.9; 1.3)	1.0 (0.8; 1.2)	1.0 (0.8; 1.2)
standing	**1.6 (1.3; 1.9)**	**2.8 (2.2; 3.4)**	**1.6 (1.3; 1.9)**	**1.6 (1.3; 2.1)**	**3.1 (2.5; 4.0)**	**1.9 (1.5; 2.3)**
dancing	**8.6 (7.0; 10.5)**	**15.6 (12.5; 19.4)**	**7.6 (6.3; 9.2)**	**7.6 (6.0; 9.6)**	**16.4 (12.9; 20.8)**	**7.9 (6.3; 9.8)**
*Compared to drawing*						
standing	**1.3 (1.1; 1.6)**	**2.0 (1.7; 2.5)**	1.1 (0.9; 1.3)	**1.5 (1.2; 1.8)**	**3.2 (2.5; 4.0)**	**1.9 (1.6; 2.4)**
dancing	**7.1 (6.0; 8.3)**	**11.7 (9.9; 13.8)**	**5.1 (4.3; 5.9)**	**7.0 (5.9; 8.3)**	**16.6 (13.6; 20.2)**	**8.2 (6.8; 10.0)**
*Compared to standing*						
dancing	**5.5 (4.4; 6.8)**	**5.7 (4.6; 7.0)**	**4.8 (3.9; 5.8)**	**4.6 (3.7; 5.8)**	**5.2 (4.2; 6.5)**	**4.3 (3.5; 5.1)**

Data in bold indicate statistical significant differences (*p* < 0.05). Activities are sorted from smallest to greatest body movement, based on the general trend for all different devices. s, seconds.

**Table A4:** Comparison of wrist-based accelerometer counts between different activities in adolescents.

Activities	Wrist Dominant *X*-axis (counts/15 s)	Wrist Dominant *Y*-axis (counts/15 s)	Wrist Dominant *Z*-axis (counts/15 s)	Wrist Non-Dominant *X*-axis (counts/15 s)	Wrist Non-Dominant *Y*-axis (counts/15 s)	Wrist Non-Dominant *Z*-axis (counts/15 s)
*Compared to watching movies*						
lying down	1.2 (0.8; 1.8)	1.3 (0.9; 2.0)	1.0 (0.5; 2.0)	1.0 (0.7; 1.4)	1.0 (0.6; 1.6)	1.3 (0.8; 2.1)
computer gaming	0.8 (0.6; 1.1)	0.9 (0.7; 1.2)	0.6 (0.3; 1.2)	0.8 (0.6; 1.1)	1.2 (0.8; 1.6)	0.9 (0.6; 1.4)
tablet use	**3.1 (2.3; 4.1)**	**2.4 (1.6; 3.6)**	**2.9 (1.6; 5.2)**	1.1 (0.8; 1.4)	0.8 (0.6; 1.1)	1.5 (1.0; 2.4)
media multitasking	**3.3 (2.4; 4.6)**	**2.4 (1.6; 3.4)**	**3.0 (1.7; 5.3)**	0.9 (0.7; 1.4)	**0.6 (0.4; 1.0)**	1.4 (0.9; 2.3)
just sitting	**2.0 (1.4; 2.8)**	**3.0 (2.2; 4.1)**	**2.7 (1.5; 4.9)**	**1.9 (1.4; 2.6)**	**2.1 (1.5; 3,0)**	**2.5 (1.5; 4.0)**
drawing	**4.4 (3.2; 6.0)**	**4.2 (2.9; 5.9)**	**7.1 (3.9; 12.7)**	**2.4 (1.8; 3.2)**	**2.0 (1.5; 2.8)**	**2.4 (1.5; 3.8)**
standing	**3.0 (2.2; 4.1)**	**5.8 (4.1; 8.2)**	**3.4 (2.0; 5.9)**	**3.0 (2.1; 4.1)**	**5.5 (3.9; 7.7)**	**3.7 (2.5; 5.6)**
dancing	**48.9 (37.7; 63.3)**	**101.1 (75.0; 136.1)**	**45.6 (26.1; 79.8)**	**31.8 (24.1; 41.9)**	**66.3 (48.8; 90.0)**	**35.5 (23.3; 54.1)**
*Compared to lying down*						
computer gaming	0.7 (0.5; 1.1)	0.7 (0.4; 1.1)	**0.6 (0.4; 1,0)**	0.8 (0.6; 1.1)	1.2 (0.7; 1.8)	0.7 (0.5; 1.0)
tablet use	**2.6 (1.8; 4.0)**	**1.9 (1.1; 3.1)**	**2.8 (1.9; 4.2)**	1.0 (0.7; 1.5)	0.8 (0.5; 1.1)	1.2 (0.8; 1.7)
media multitasking	**2.9 (1.9; 4.3)**	**1.8 (1.2; 2.9)**	**2.9 (2.0; 4.4)**	0.9 (0.6; 1.5)	0.6 (0.4; 1.1)	1.1 (0.7; 1.7)
just sitting	**1.7 (1.2; 2.5)**	**2.3 (1.5; 3.6)**	**2.6 (1.8; 3.7)**	**1.9 (1.3; 2.6)**	**2.1 (1.4; 3.2)**	**1.9 (1.3; 2.8)**
drawing	**3.8 (2.5; 5.7)**	**3.2 (2.0; 5.2)**	**6.9 (4.5; 10.7)**	**2.4; (1.7; 3.3)**	**2.0 (1.3; 3.0)**	**1.9 (1.2; 2.8)**
standing	**2.6 (1.7; 3.9)**	**4.5 (2.8; 7.2)**	**3.4 (2.2; 5.2)**	**2.9 (2.1; 3.9)**	**5.4 (3.6; 8.0)**	**2.9 (2.0; 4.2)**
dancing	**42.2 (28.9; 61.6)**	**78.1 (50.2; 121.6)**	**44.8 (30.1; 66.5)**	**31.2 (22.5; 43.2)**	**65.4 (44.6; 95.9)**	**27.3 (18.7; 39.9)**
*Compared to computer gaming*						
tablet use	**3.8 (2.9; 5.0)**	**2.7 (2.0; 3.7)**	**4.7 (3.4; 6.4)**	1.3 (0.9; 1.9)	**0.7 (0.5; 0.9)**	**1.7 (1.3; 2.2)**
media multitasking	**4.1 (3.0; 5.6)**	**2.6 (1.8; 3.8)**	**4.9 (3.4; 7.0)**	1.2 (0.9; 1.6)	**0.6 (0.4; 0.8)**	**1.6 (1.2; 2.1)**
just sitting	**2.5 (1.8; 3.5)**	**3.4 (2.4; 4.7)**	**4.4 (2.9; 6.5)**	**2.4 (1.8; 3.2)**	**1.8 (1.3; 2.7)**	**2.7 (1.9; 3.8)**
drawing	**5.4 (4.0; 7.4)**	**4.7 (3.4; 6.4)**	**11.6 (7.9; 17.0)**	**3.0 (2.4; 3.8)**	**1.7 (1.3; 2.3)**	**2.7 (2.0; 3.5)**
standing	**3.7 (2.8; 4.9)**	**6.5 (4.8; 8.8)**	**5.6 (3.7; 8.5)**	**3.7 (2.8; 4.9)**	**4.7 (3.3; 6.7)**	**4.1 (3.0; 5.6)**
dancing	**60.6 (46.9; 78.3)**	**113.5 (85.2; 151.0)**	**74.4 (52.8; 105.5)**	**39.7 (32.4; 48.6)**	**56.8 (43.1; 75.1)**	**38.9 (30.0; 50.4)**
*Compared to tablet use*						
media multitasking	1.1 (0.9; 1.3)	1.0 (0.7; 1.3)	1.0 (0.8; 1.3)	0.9 (0.6; 1.3)	0.8 (0.5; 1.3)	0.9 (0.7; 1.2)
just sitting	**0.7 (0.5; 0.9)**	1.2 (0.8; 1.8)	0.9 (0.7; 1.3)	**1.8 (1.3; 2.5)**	**2.8 (1.9; 4.1)**	**1.6 (1.2; 2.1)**
drawing	**1.4 (1.1; 1.8)**	**1.7 (1.3; 2.4)**	**2.5 (1.9; 3.2)**	**2.3 (1.6; 3.3)**	**2.6 (1.8; 3.7)**	**1.6 (1.1; 2.1)**
standing	1.0 (0.7; 1.3)	**2.4 (1.7; 3.4)**	1.2 (0.9; 1.6)	**2.8 (1.9; 4.1)**	**7.1 (4.8; 10.5)**	**2.4 (1.8; 3.2)**
dancing	**16.0 (13.6; 18.6)**	**41.7 (32.0; 54.4)**	**16.0 (12.7; 20.2)**	**29.9 (21.0; 42.5)**	**85.6 (59.7; 122.7)**	**23.1 (17.3; 30.9)**
*Compared to Media multitasking*						
just sitting	**0.6 (0.5; 0.8)**	1.3 (0.9; 1.8)	0.9 (0.7; 1.2)	**2.0 (1.4; 2.8)**	**3.3 (2.3; 4.9)**	**1.7 (1.3; 2.4)**
drawing	**1.3 (1.1; 1.7)**	**1.8 (1.4; 2.3)**	**2.4 (1.9; 3.0)**	**2.5 (1.8; 3.6)**	**3.1 (2.1; 4.5)**	**1.7 (1.2; 2.3)**
standing	0.9 (0.6; 1.3)	**2.5 (1.7; 3.5)**	1.1 (0.8; 1.6)	**3.1 (2.2; 4.5)**	**8.5 (5.7; 12.7)**	**2.6 (1.9; 3.5)**
dancing	**14.8 (12.3; 17.7)**	**43.0 (34.9; 52.9)**	**15.3 (12.6; 18.6)**	**33.4 (24.3; 46.0)**	**102.6 (69.3; 151.8)**	**24.7 (18.4; 33.3)**
*Compared to just sitting*						
drawing	**2.2 (1.6; 3.0)**	**1.4 (1.0; 1.9)**	**2.6 (2.0; 3.6)**	1.3 (0.9; 1.7)	0.9 (0.7; 1.3)	1.0 (0.7; 1.4)
standing	**1.5 (1.1; 2.0)**	**1.9 (1.4; 2.6)**	1.3 (1.0; 1.7)	**1.6 (1.2; 2.1)**	**2.6 (1.9; 3.4)**	**1.5 (1.2; 1.9)**
dancing	**24.4 (19.0; 31.3)**	**33.6 (25.7; 44.0)**	**17.1 (13.3; 21.8)**	**16.7 (13.1; 21.4)**	**31.0 (23.7; 40.4)**	**14.3 (11.0; 18.9)**
*Compared to drawing*						
standing	**0.7 (0.5; 0.9)**	**1.4 (1.0; 1.8)**	**0.5 (0.4; 0.7)**	1.2 (0.9; 1.6)	**2.7 (2.1; 3.6)**	**1.5 (1.1; 2.1)**
dancing	**11.1 (9.2; 13.4)**	**24.2 (20.1; 29.2)**	**6.5 (5.5; 7.6)**	**13.1 (10.6; 16.3)**	**32.8 (26.1; 41.3)**	**14.7 (11.6; 18.6)**
*Compared to standing*						
dancing	**16.4 (12.6; 21.3)**	**17.5 (13.4; 22.8)**	**13.4 (10.3; 17.3)**	**10.7 (8.6; 13.5)**	**12.1 (9.7; 15.1)**	**9.6 (7.7; 11.9)**

Data in bold indicate statistical significant differences (*p* < 0.05). Activities are sorted from smallest to greatest body movement, based on the general trend for all different devices. s, seconds.

**Table A5:** Comparison of leg muscle activity between different activities in children and adolescents.

Activities	Child			Adolescent		
	Muscle Activity Vastus Lateralis *	Muscle Activity Rectus Femoris *	Muscle Activity Gastrocnemius Medialis *	Muscle Activity Vastus Lateralis *	Muscle Activity Rectus Femoris *	Muscle activity Gastrocnemius Medialis *
*Compared to watching movies*						
lying down	−1.2 (−3.2; 0.7)	−2.6 (−9.1; 4.0)	−0.2 (−1.1; 0.8)	−0.8 (−2.4; 0.8)	−0.8 (−1.8; 0.3)	−0.1 (−0.6; 0.3)
computer gaming	−0.5 (−2.2; 1.1)	−1.6 (−8.0; 4.8)	1.1 (−0.7; 3.0)	−0.1 (−0.8; 0.6)	1.2 (−0.4; 2.9)	1.4 (−0.2; 3.1)
tablet use	−1.4 (−3.3; 0.4)	−2.7 (−9.2; 3.8)	−0.1 (−1.0; 0.7)	−0.5 (−1.8; 0.8)	−0.5 (−1.5; 0.5)	0.7 (−0.2; 1.6)
media multitasking	−0.6 (−2.7; 1.5)	−1.4 (−8.5; 5.8)	0.4 (−0.5; 1.3)	0.8 (0.0; 1.6)	0.5 (−0.8; 1.8)	0.6 (−0.1 1.3)
just sitting	−0.6 (−2.7; 1.5)	−1.4 (−8.2; 5.5)	−0.9 (−2.0; 0.1)	**2.5 (0.2; 4.9)**	**1.6 (0.3; 2.9)**	0.3 (−0.3; 0.9)
drawing	−1.4 (−3.3; 0.4)	−3.0 (−9.6; 3.6)	**−0.8 (−1.7; 0.0)**	−0.3 (−0.7; 0.2)	0.2 (−0.5; 0.9)	0.3 (−0.3; 1.0)
standing	0.4 (−1.7; 2.5)	−1.7 (−8.3; 4.9)	**2.8 (1.8; 3.8)**	2.0 (−0.3; 4.3)	0.9 (−0.5; 2.2)	**3.3 (2.2; 4.3)**
dancing	**6.9 (3.9; 9.9)**	**8.0 (1.1; 14.9)**	**7.0 (5.4; 8.6)**	**15.5 (9.8; 21.3)**	**18.7 (10.1; 27.3)**	**14.5 (9.9; 19.1)**
*Compared to lying down*						
computer gaming	0.7 (−0.1; 1.5)	1.0 (−0.4; 2.4)	1.3 (−0.6; 3.2)	0.7 (−0.3; 1.7)	2.0 (0.6; 3.4)	1.6 (0.2; 3.0)
tablet use	−0.2 (−0.4; 0.1)	−0.1 (−0.9; 0.6)	0.0 (−0.9; 1.0)	0.3 (−0.1; 0.8)	0.3 (−0.1; 0.6)	0.9 (0.0; 1.7)
media multitasking	−0.3 (−0.7; 0.0)	1.2 (−1.5; 3.9)	0.6 (−0.2; 1.4)	1.6 (−0.4; 3.6)	1.3 (0.3; 2.3)	0.7 (0.2; 1.3)
just sitting	0.6 (0.0; 1.2)	**1.2 (0.2; 2.3)**	**−0.8 (−1.4; −0.1)**	**3.3 (0.2; 6.4)**	**2.3 (0.5; 4.2)**	**0.4 (0.2; 0.7)**
drawing	−0.1 (−0.4; 0.1)	−0.4 (−1.0; 0.2)	**−0.7 (−1.3; −0.1)**	0.5 (−0.7; 1.7)	1.0 (−0.5; 2.5)	0.5 (−0.1; 1.1)
standing	**1.7 (1.0; 2.3)**	0.9 (−0.1; 1.8)	**3.0 (1.8; 4.2)**	**2.8 (1.5; 4.2)**	**1.6 (0.9; 2.3)**	**3.4 (2.3; 4.5)**
dancing	**8.2 (6.2; 10.2)**	**10.6 (6.7; 14.4)**	**7.2 (5.3; 9.1)**	**16.3 (9.1; 23.5)**	**19.4 (10.5; 28.4)**	**14.6 (10.0; 19.3)**
*Compared to computer gaming*						
tablet use	**−0.9 (−1.7; 0.0)**	**−1.1 (−2.1; −0.1)**	−1.3 (−3.1; 0.6)	−0.3 (−1.0; 0.3)	**−1.7 (−3.1; −0.3)**	−0.7 (−2.2; 0.8)
media multitasking	**−1.0 (−1.9; −0.1)**	0.2 (−2.6; 3.1)	−0.7 (−2.7; 1.2)	0.9 (−0.3; 2.2)	−0.7 (−1.7; 0.3)	−0.8 (−1.8; 0.1)
just sitting	−0.1 (−1.1; 0.9)	0.2 (−1.2; 1.7)	**−2.1 (−3.9; −0.2)**	**2.6 (0.1; 5.2)**	0.3 (−1.1; 1.8)	−1.1 (−2.3; 0.0)
drawing	**−0.8 (−1.6; 0.0)**	**−1.4 (−2.5; −0.3)**	**−2.0 (−3.8; −0.2)**	−0.1 (−0.5; 0.2)	−1.0 (−2.7; 0.6)	−1.1 (−2.7; 0.5)
standing	1.0 (−0.2; 2.1)	−0.1 (−1.2; 0.9)	1.7 (−0.4; 3.8)	**2.1 (0.3; 3.9)**	−0.4 (−1.6; 0.8)	**1.8 (0.0; 3.7)**
dancing	**7.5 (5.1; 9.9)**	**9.6 (5.8; 13.4)**	**5.9 (4.2; 7.6)**	**15.6 (9.3; 22.0)**	**17.4 (8.9; 25.9)**	**13.1 (7.9; 18.3)**
*Compared to tablet use*						
media multitasking	−0.1 (−0.4; 0.1)	1.3 (−1.3; 3.9)	0.5 (−0.2; 1.3)	1.3 (−0.3; 2.9)	1.0 (0.0; 2.1)	−0.1 (−1.0; 0.7)
just sitting	**0.8 (0.1; 1.6)**	**1.3 (0.2; 2.5)**	−0.8 (−1.8; 0.2)	**3.0 (0.2; 5.8)**	**2.1 (0.3; 3.9)**	−0.4 (−1.2; 0.3)
drawing	**0.1 (−0.3; 0.4)**	**−0.3 (−0.8; 0.2)**	**−0.7 (−1.5; 0.1)**	**0.2 (−0.7; 1.1)**	**0.7 (−0.7; 2.1)**	**−0.4(−1.5; 0.7)**
standing	**1.8 (1.2; 2.5)**	**1.0 (0.4; 1.5)**	**3.0 (2.0; 3.9)**	**2.5 (1.0; 3.9)**	**1.4 (0.7; 2.0)**	**2.6 (1.1; 4.0)**
dancing	8.4 (6.4; 10.3)	10.7 (7.2; 14.2)	7.2 (5.4; 9.0)	16.0 (9.1; 22.8)	19.2 (10.2; 28.1)	13.8 (8.7; 18.9)
*Compared to Media multitasking*						
just sitting	**0.9 (0.1; 1.8)**	0.0 (−2.9; 2.9)	**−1.3 (−2.0; −0.6)**	1.7 (−0.7; 4.1)	1.0 (−0.4; 2.5)	−0.3 (−0.7; 0.1)
drawing	0.2 (−0.2; 0.6)	−1.6 (−4.1; 0.9)	**−1.2 (−1.9; −0.6)**	**−1.1 (−2.1; 0.0)**	−0.3 (−1.6; 1.0)	−0.2 (−1.1; 0.6)
standing	**2.0 (1.3; 2.7)**	−0.4 (−2.7; 2.0)	**2.4 (1.5; 3.4)**	**1.2 (−1.4; 3.8)**	0.3 (−0.6; 1.3)	2.7 (1.6; 3.8)
dancing	**8.5 (6.5; 10.5)**	**9.3 (4.9; 13.8)**	**6.7 (4.9; 8.4)**	**14.7 (9.1; 20.3)**	**18.1 (9.4; 26.8)**	13.9 (9.1; 18.7)
*Compared to just sitting*						
drawing	**−0.7 (−1.5; 0.0)**	**−1.6 (−2.9; −0.4)**	0.1 (−0.3; 0.5)	**−2.8 (−5.2; −0.3)**	**−1.4 (−2.2; −0.5)**	0.1 (−0.7; 0.8)
Standing	**1.0 (0.3; 0.2)**	−0.4 (−1.7; 1.0)	**3.8 (2.7; 4.8)**	−0.5 (−3.4; 2.4)	−0.7 (−2.6; 1.1)	**3.0 (1.8; 4.2)**
dancing	**7.6 (5.6; 9.5)**	**9.3 (5.7; 13.0)**	**8.0 (6.2; 9.7)**	**13.0 (7.6; 18.4)**	**17.1 (9.1; 25.1)**	**14.2 (9.5; 18.9)**
*Compared to drawing*						
standing	**1.8 (1.0; 2.5)**	**1.3 (0.6; 1.9)**	**3.7 (2.8; 4.5)**	**2.3 (0.3; 4.3)**	0.7 (−1.0; 2.3)	**2.9 (1.8; 4.1)**
dancing	**8.3 (6.2; 10.4)**	**11.0 (7.2; 14.8)**	**7.9 (6.3; 9.5)**	**15.8 (9.7; 21.9)**	**18.5 (10.2; 26.7)**	**14.2 (9.7; 18.6)**
*Compared to standing*						
dancing	**6.5 (4.8; 8.3)**	**9.7 (6.0; 13.5)**	**4.2 (2.8; 5.6)**	**13.5 (6.1; 20.9)**	**17.8 (8.8; 26.8)**	**11.2 (6.8; 15.7)**

* expressed as a percentage of the 95th percentile of EMG values during stepping. Data in bold indicate statistical significant differences (*p* < 0.05). Activities are sorted from smallest to greatest body movement, based on the general trend for all different devices.

**Table A6:** Comparison of heart rate between different activities in children and adolescents.

Activities	Children	Adolescents
	Heart Rate (beats/minute)	Heart Rate (beats/minute)
*Compared to watching movies*		
lying down	−0.7 (−2.3; 1.0)	−1.2 (−3.1; 0.8)
computer gaming	**1.9 (0.3; 3.6)**	**4.9 (2.7; 7.1)**
tablet use	**2.4 (0.9; 4.0)**	1.4 (−0,2; 3.1)
media multitasking	**4.7 (2.7; 6.8)**	**3.3 (1.4; 5.2)**
just sitting	**7.4 (5.6; 9.3)**	**11.7 (9.4; 14.0)**
drawing	**6.8 (5.4; 8.2)**	**7.1 (5.3; 9.0)**
standing	**18.1 (15.5; 20.6)**	**21.0 (18.6; 23.5)**
dancing	**34.1 (29.4; 38.7)**	**45.6 (40.0; 51.1)**
*Compared to lying down*		
computer gaming	**2.6 (0.8; 4.4)**	**6.0 (3.5; 8.5)**
tablet use	**3.1 (1.5; 4.7)**	**2.6 (0.3; 4.9)**
media multitasking	**5.4 (3.7; 7.1)**	**4.4 (2.2; 6.7)**
just sitting	**8.1 (6.2; 10.0)**	**12.8 (10.3; 15.4)**
drawing	**7.5 (5.8; 9.2)**	**8.3 (5.9; 10.7)**
standing	**18.7 (16.5; 20.9)**	**22.2 (19.4; 25.0)**
dancing	**34.7 (30.4; 39.0)**	**46.7 (41.4; 52.1)**
*Compared to computer gaming*		
tablet use	0.5 (−1.4; 2.4)	**−3.4 (−6.0; −0.9)**
media multitasking	**2.8 (1.0; 4.6)**	−1.6 (−3.9; 0.7)
just sitting	**5.5 (3.8; 7.2)**	**6.8 (4.5; 9.1)**
drawing	**4.9 (3.3; 6.4)**	**2.3 (0.5; 4.0)**
standing	**16.1 (13.8; 18.5)**	**16.2 (13.4; 18.9)**
dancing	**32.1 (27.7; 36.5)**	**40.7 (35.3; 46.1)**
*Compared to tablet use*		
media multitasking	**2.3 (0.6; 3.9)**	1.9 (−0.2; 4.0)
just sitting	**5.0 (3.0; 7.0)**	**10.3 (7.7; 12.8)**
drawing	**4.4 (2.7; 6.0)**	**5.7 (3.6; 7.8)**
standing	**15.6 (13.5; 17.8)**	**19.6 (17.0; 22.3)**
dancing	**31.6 (27.2; 36.0)**	**44.2 (38.4; 49.9)**
*Compared to media multitasking*		
just sitting	**2.7 (1.2; 4.2)**	**8.4 (7.2; 9.6)**
drawing	**2.1 (0.3; 3.9)**	**3.8 (1.8; 5.9)**
standing	**13.3 (11.4; 15.2)**	**17.8 (15.4; 20.1)**
dancing	**29.3 (25.7; 33.0)**	**42.3 (37.6; 47.0)**
*Compared to just sitting*		
drawing	−0.6 (−2.0; 0.8)	**−4.5 (−6.9; −2.1)**
Standing	**10.6 (8.3; 13.0)**	**9.4 (6.9; 11.8)**
dancing	**26.6 (22.5; 30.8)**	**33.9 (29.3; 38.5)**
*Compared to drawing*		
standing	**11.2 (8.8; 13.7)**	**13.9 (11.6; 16.2)**
dancing	**27.2 (22.6; 31.9)**	**38.4 (33.1; 43.8)**
*Compared to standing*		
dancing	**16.0 (12.1; 19.9)**	**24.5 (19.1; 30.0)**

Data in bold indicate statistical significant differences (*p* < 0.05). Activities are sorted from smallest to greatest body movement, based on the general trend for all different devices.
